# “BAX602” in Preventing Surgical Adhesion after Extracorporeal Ventricular Assist Device Implantation for Refractory Congestive Heart Failure: Study Protocol for a Multicenter Randomized Clinical Trial

**DOI:** 10.1007/s10557-020-06990-2

**Published:** 2020-05-22

**Authors:** Satsuki Fukushima, Koko Asakura, Toshimitsu Hamasaki, Kaori Onda, Takuya Watanabe, Akira Shiose, Minoru Ono, Norihide Fukushima, Haruko Yamamoto, Tomoyuki Fujita

**Affiliations:** 1 Department of Cardiac Surgery, National Cerebral and Cardiovascular Center, 6-1 Kishibeshimmachi, Suita, Osaka, 564-8565 Japan; 2 Department of Data Science, National Cerebral and Cardiovascular Center, Osaka, Japan; 3 The Biostatistics Center and Department of Biostatistics and Bioinformatics, George Washington University, Washington, DC USA; 4 Department of Transplant Medicine, National Cerebral and Cardiovascular Center, Osaka, Japan; 5 Department of Cardiovascular Surgery, Kyushu University Hospital, Fukuoka, Japan; 6 Department of Cardiac Surgery, The University of Tokyo, Tokyo, Japan

**Keywords:** Adhesion prevention device, Cardiac surgery, Ventricular assist device, Multicenter randomized trial

## Abstract

**Background:**

The high surgical risk in redo cardiac surgery is largely attributed to adhesions around the epicardium and the great vessels. BAX602 is an adhesion prevention reagent composed of two synthetic polyethylene glycols. Spraying BAX602 over the epicardium and the great vessels reportedly contributes to adhesion prevention after pediatric cardiac surgery. The present study aims to evaluate the safety and effectiveness of BAX602 spray in patients undergoing extracorporeal ventricular assist device implantation surgery to treat refractory congestive heart failure.

**Methods and Design:**

This investigator-initiated, multicenter, pivotal, two-arm, open-label, randomized trial will include a total of 30 patients. The primary outcome measure is the severity of adhesions, which will be evaluated during re-sternotomy surgery performed 2–12 weeks after the primary extracorporeal ventricular assist device implantation surgery. The adhesion severity will be evaluated at five predefined sites using a four-grade adhesion evaluation score (0 = no adhesion; 1 = filmy and avascular adhesion; 2 = dense/vascular adhesion; 3 = cohesive adhesion). This measure will be summarized in two ways to evaluate the effect of BAX602: (1) the total score of the severity of adhesions at all five sites (ranging from 0 to 15), and (2) the total number of sites with dense/vascular or cohesive adhesions (ranging from 0 to 5).

**Ethics and Dissemination:**

The study findings will be disseminated at regional, national, and international conferences and through peer-reviewed scientific journals.

**Trial Registration:**

The trial was registered in the UMIN Clinical Trials Registry (UMIN-CTR: UMIN000038998) on 6 January 2020**.**

## Background

Redo cardiac surgery by re-entry through the sternum carries a substantial surgical risk that is two times greater than the risk associated with the primary cardiac surgery [1–4]. The high surgical risk in redo cardiac surgery is largely attributed to adhesions around the epicardium and the great vessels, which prolongs the cardiopulmonary bypass time and total operation time and increases blood product use, inducing postoperative complications [4, 5]. In particular, the degree of intrapericardial and mediastinal adhesion influences the clinical outcome of redo surgery in patients with an implanted ventricular assist device (VAD) [6]. This increased degree of adhesion after VAD implantation occurs because adhesion is more severe around prosthetic material than native tissue, and the coagulopathic state associated with the VAD exacerbates bleeding during the adhesiolysis, consequently deteriorating the hemodynamic state. Furthermore, in bridge-to-transplantation surgery, the time spent on adhesiolysis limits the time allowed for organ procurement and preservation.

Tissue adhesion after surgical procedures has been well studied at the cellular and molecular levels [7]. Adhesion is initiated just after the surgical procedures, as macrophages are initially recruited from the circulating blood to the surgically exposed areas. Subsequently, settled and activated macrophages form the basis of adhesion by releasing extracellular matrices within 3 days, and angiogenic activities are launched from this basis. Within 5 days, a new vessel network is integrated between the tissues to form structured adhesion tissue [8]. While the structured adhesion tissues are gradually cemented during the subsequent 2 months, new adhesion tissues are not formed after 7 days postoperatively [7, 9]. It is thus suggested that inhibition of the recruitment, settlement, and activation of macrophages to the surgically exposed area for at least 7 days may substantially contribute to the prevention of adhesion tissue formation.

Several adhesion prevention reagents have been proposed. The application of Coseal spray (Baxter Healthcare Corporations, Deerfield IL, USA), hereafter referred to as BAX602, over the epicardium and the great vessels reportedly contributes to adhesion prevention after surgical procedures, including cardiac surgery [10, 11] and even VAD implantation surgery [12, 13]. BAX602 is composed of two synthetic polyethylene glycols (PEGs): a dilute hydrogen chloride solution and a sodium phosphate/sodium carbonate solution. When these two solutions mix, they polymerize to form a hydrogel that inhibits adhesion formation. Spraying the heart and the great vessels with BAX602 causes this hydrogel to coat the surface of the heart and the great vessels; this hydrogel coating is sustained for 7 days before being hydrolyzed, consequently preventing adhesion formation over the surface of the heart and the great vessels [11]. Although there are positive reports of the use of BAX602 in pediatric cardiac surgery in observational [11] and prospective randomized [10] studies, the safety and efficacy of BAX602 for adhesion prevention have not been fully established in adult patients with an implanted VAD.

Because no devices have been approved in Japan for tissue adhesion prevention of thoracic organs, including cardiac surgical cases, this investigator-initiated trial of BAX602 was designed as a multicenter phase I/II randomized controlled study. The aim of this proposed study is to evaluate the safety and effectiveness of spraying BAX602 over the surface of the heart and great vessels for adhesion prevention in patients undergoing extracorporeal VAD implantation surgery to treat refractory congestive heart failure. The subsequent goal of this study is the approval of BAX602 for adhesion prevention in cardiac surgery in Japan.

## Methods and Analysis

### Study Purpose

The present study aims to evaluate the effects of BAX602 in preventing adhesions around the epicardium and the great vessels in patients undergoing extracorporeal VAD implantation for refractory congestive heart failure.

### Study Setting

**Fig. 1:**
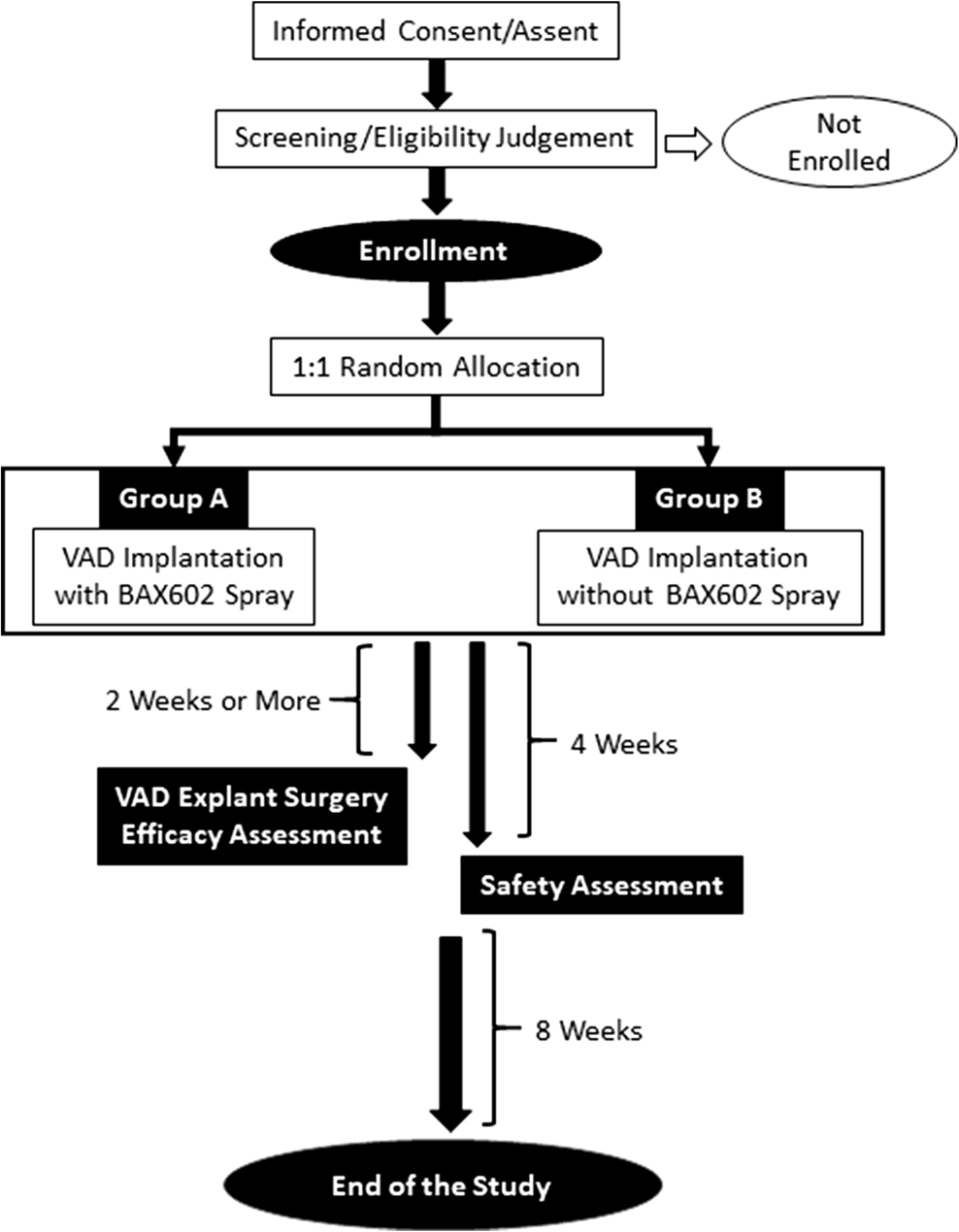
Summary of the Study Design. VAD, ventricular assist device

The study is an investigator-initiated, multicenter, pivotal, two-arm, open-label, randomized trial. The flowchart of the trial is shown in Fig. 1.

### Assessment of Adhesion

Pericardial adhesion will be macroscopically evaluated during VAD explant surgery performed 2–12 weeks after the primary surgery. The VAD explant surgery is performed to either wean the patient from the VAD as a result of sufficient cardiac functional recovery or to exchange the initial VAD with a durable VAD as a result of insufficient recovery [14]. Patients who undergo VAD explant surgery 1–13 days after the primary thoracotomy will be excluded from the efficacy assessment.

**Fig. 2:**
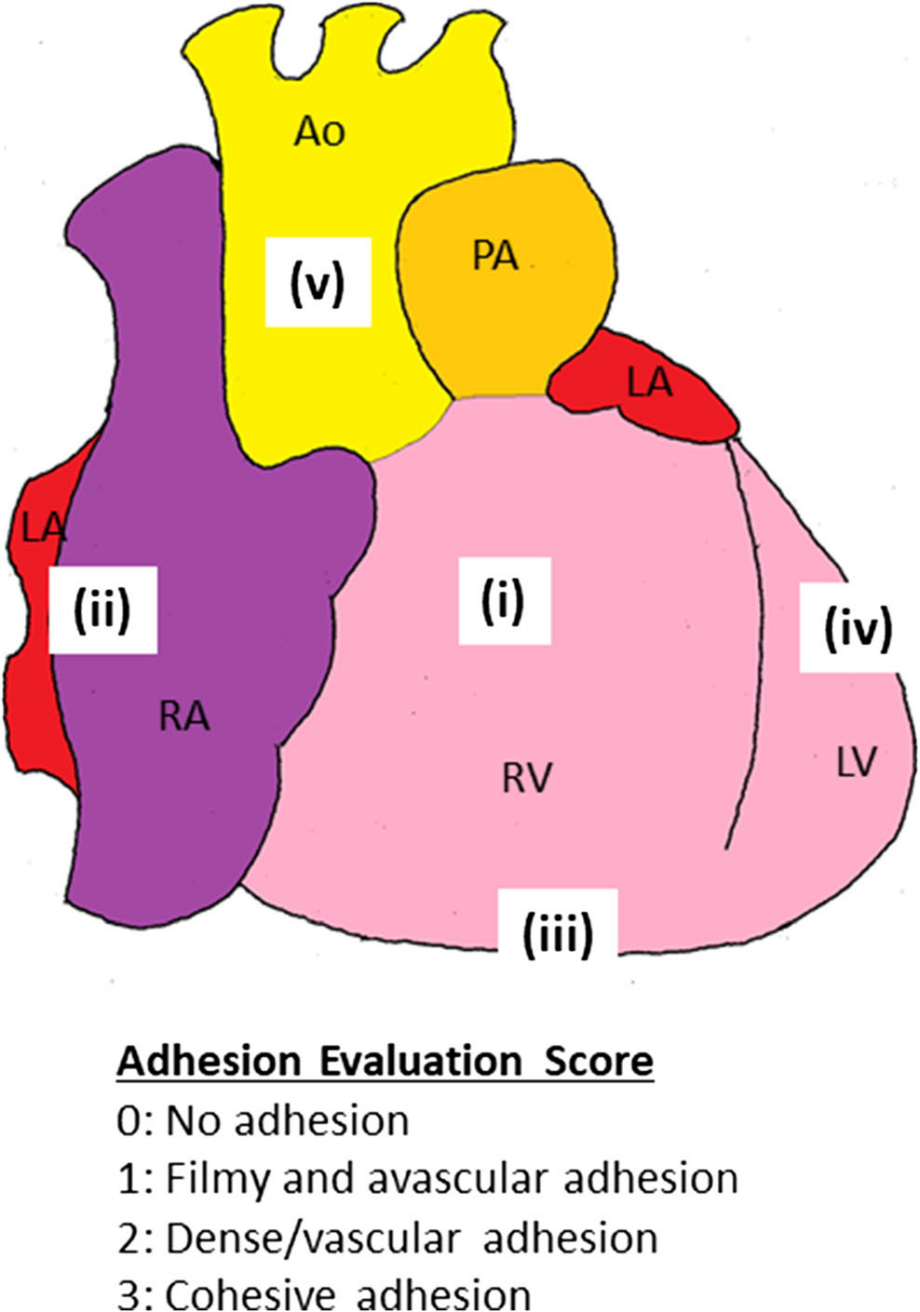
Evaluation of pericardial adhesion. Pericardial adhesion will be evaluated at the VAD explant surgery performed 2–12 weeks after the primary extracorporeal VAD implantation surgery. The five sites at which the severity of adhesion will be evaluated using a four-grade adhesion evaluation score are: (i) anterior surface of the RV, (ii) right lateral surface of the RA, (iii) diaphragmatic surface, (iv) left lateral surface of the LV, and (v) surface of the ascending Ao. VAD, ventricular assist device; Ao, aorta; PA, pulmonary artery; LA, left atrium; RA, right atrium; LV, left ventricle; RV, right ventricle

The adhesion severity will be evaluated at the following five predefined sites: (i) anterior surface of the right ventricle, (ii) right lateral surface of the right atrium, (iii) diaphragmatic surface, (iv) left lateral surface of the left ventricle, and (v) surface of the ascending aorta. Adhesion severity will be evaluated using a four-grade scoring system where 0 = no adhesion, 1 = filmy and avascular adhesion, 2 = dense/vascular adhesion, and 3 = cohesive adhesion (Fig. 2) [11].

### Study Endpoints

The primary outcome measure is the severity of adhesions at the five predefined sites (Fig. 2). This measure will be summarized in the following two ways: (1) total score of the severity of adhesions across all five sites, ranging from 0 (no adhesions at any of the five sites) to 15 (cohesive adhesions at all five sites); (2) total number of sites with dense/vascular or cohesive adhesions, ranging from 0 (no adhesions or filmy and avascular adhesions at any of the five sites) to 5 (dense/vascular or cohesive adhesions at all five sites). These two summary measures will serve as multiple primary endpoints. As adhesion formation generally begins immediately after surgery, and no new adhesion formation occurs after 1 week postoperatively, the severity of adhesions measured at 2 weeks or more after the primary thoracotomy will be used in the primary analysis of the two primary endpoints [15].

The secondary endpoints are: (1) total score of the severity of adhesions across all five sites at 1–12 weeks after the primary thoracotomy, (2) total number of sites with dense/vascular or cohesive adhesions at 1–12 weeks after the primary thoracotomy, (3) survival at the end of the observation period, (4) re-thoracotomy for hemostasis after primary thoracotomy, (5) transfusion volume during re-thoracotomy, and (6) absence of mediastinitis requiring surgical intervention.

### Patient Selection

Inclusion and exclusion criteria are shown in Table 1. In summary, the study will include patients undergoing extracorporeal VAD implantation surgery to treat refractory congestive heart failure that needs mechanical circulatory with or without pulmonary support.

**Table 1:** Study inclusion and exclusion criteria

Inclusion criteria	
Patients may enter the trial if all of the following apply:	
1. Age 12 years or older and younger than 80 when providing consent.	
2. To receive an extracorporeal ventricular assist device to treat acute cardiogenic circulatory failure.	
3. Written informed consent from the patient or legal representative/close relative	
Exclusion criteria	
Patients may not enter the trial if any of the following apply:	
1. History of cardiac or great vessel surgery	
2. Participating in another clinical trial at time of enrolment	
3. Deemed unsuitable by the primary investigator for other reasons	

### Group Allocation

Eligible patients will be registered and randomly assigned in a 1:1 ratio to either the surgery with BAX602 group (group A) or the surgery-alone group (group B), using permutation block randomization stratified by site. To ensure concealment, the block sizes will not be disclosed. Owing to the nature of the intervention, the investigators cannot be blinded to the allocations. Assessments regarding adhesion severity will be conducted by the operator of the re-thoracotomy in the presence of the cardiothoracic surgeon who is not involved in the primary thoracotomy. Video footage of the surgical field will be collected during re-thoracotomy.

### Trial Device

BAX602 is not approved in Japan, although it has been approved and is being sold in several Western countries. BAX602 is composed of three parts: the syringe pre-filled with the spray solution, the product-specific gas-driven spray device, and the spray kit that includes the tube and spray head. The spray solution is composed of two synthetic PEGs (a dilute hydrogen chloride solution and a sodium phosphate/sodium carbonate solution) provided in kits of 8 mL.

### Treatment Methods

The treatment flowchart is shown in Fig. 1. Surgery will be performed by the median sternotomy approach in all patients in both groups, with the inflow of the VAD being the LV apex and the outflow being the ascending aorta with or without the main pulmonary artery. In group A, prior to sternal closure, the spray device and the spray kit will be used to spray 8 mL of BAX602 in a thin homogeneous layer on the mediastinum, covering the visible surface of the heart and great vessels. In group B, no agent will be applied prior to sternal closure.

### Follow-Up

All patients will be followed up to detect any adverse events for 4 weeks after the primary surgery or until re-thoracotomy, whichever is longer. Follow-up will end at 12 weeks after the primary surgery of the last patient (last patient-out). The study schedule is shown in Table 2. The second surgery, in which the degree of pericardial adhesion will be assessed, will be performed via the re-median sternotomy approach.

**Table 2:** Schedule of evaluation during the study

	Screening	Primary thoracotomy		4 weeks after the thoracotomy	Re-thoracotomy
		before	after		
Inclusion/exclusion criteria	X				
Signed consent form	X				
Medical/treatment history	X				
Vital signs		X			
Cardiac echography	X				X
Chest Xray	X				X
Laboratory tests	X			X	X
Surgical procedure of the primary thoracotomy			X	X	
Surgical procedure of the re-thoracotomy					X
Medication			X	X	X
Adverse events					
Survival				X	X

### Sample Size

The sample size was calculated based on the total adhesion severity score, assuming a mean difference in total score of 5.0 between the two groups and a standard deviation of 3.5, referring to published data [11]. Thus, 12 patients are required in each group, assuming a two-sided t-test with *α* = 0.05 with a power of 80%, and a simple Bonferroni correction to control the type I error rate due to the existence of two primary endpoints (a total alpha of 0.05 was equally allocated to each endpoint, i.e., *α* = 0.025). Based on the simulation, in most scenarios that were considered, a sample size of 12 in each group provided at least 80% power to detect a frequency difference in the total number of sites with dense/vascular or cohesive adhesions across all five sites, using the two-sided Fisher’s exact test at α = 0.025. To allow for a drop-out rate of 30% for the primary analysis of the primary endpoints, 15 patients will be recruited per group, i.e., 30 patients in total.

### Statistical Methods

Analysis will be based on the intention-to-treat principle. Continuous data will be presented as mean ± standard deviation or median and range. Categorical data will be presented as frequencies with proportions (%). Group A will be compared with group B for all primary and secondary analyses. For intergroup comparisons, Fisher’ exact test will be used for categorical variables, while Welch’s t-test or Wilcoxon’s rank sum test will be used for continuous variables. In addition, the differences between means or proportions for continuous or categorical variables will be calculated with corresponding 95% confidence intervals, where appropriate.

The two primary endpoints will be analyzed by the Bonferroni–Holm procedure, with equal *α* allocation to control the type I error rate; both endpoints will first be tested at α = 0.025. If one of the endpoints significantly differs between groups but the other does not, the other endpoint will be tested again at the α = 0.05 level.

For the secondary endpoints, categorical variables will be assessed with the chi-square test or Fisher’s exact test, while continuous variables will be assessed with the t-test or Wilcoxon’s rank-sum test, as appropriate. For time-to-event variables, the event-free survival curve will be estimated using the Kaplan–Meier method and will be compared between the two groups using the log-rank test. The hazard ratio with its 95% confidence interval will be calculated using a proportional hazards model.

The analyses will be conducted using the most recent version of SAS (Cary, NC, USA). All tests will be two-sided. The statistical analysis plan, which includes a more technical and detailed elaboration of the principal features stated in the present protocol, will be prepared separately and finalized before the database is locked.

### Data Management, Monitoring, and Auditing

When each patient is enrolled, data will be obtained using the Good Clinical Practice standards. The system for electronic data capture and data management has been validated to meet the Japanese regulatory requirements. On-site monitoring is planned, including source document verification and auditing.

### Data and Safety Monitoring Committee

The Data and Safety Monitoring Committee is composed of three individuals who will not be involved in conducting the study and have expertise in cardiovascular diseases and open-heart surgery, including a cardiovascular surgeon and two cardiovascular physicians. The Data and Safety Monitoring Committee will independently review the reports regarding the safety data derived from this study.

### Participating Institutions

Three Japanese institutions are expected to participate in this study, including the National Cerebral Cardiovascular Center Hospital, University of Tokyo Hospital, and Kyushu University Hospital.

### Trial Status

The protocol number of this study is NCVC-BAX602 version 2.0 (November 28, 2019). Patient enrollment began in January 2020 and will be terminated in May 2021. The study will be completed in August 2021.

## Discussion

Several products for adhesion prevention are currently under development and in clinical use, represented by Seprafilm (Baxter Healthcare Corporations) [16]. The adhesion prevention effects of BAX602 are similar to these products; however, BAX602 differs from other products in the following aspects. First, BAX602 is a PEG-based product, while Seprafilm is composed of sodium hyaluronate and carboxylmethylcellulose. Second, BAX602 is a hydrogel applied to the target organ surface by spray application, while Seprafilm is a membrane. As the cardiac surface contracts, a membrane may not be appropriately settled over the surface, while the BAX602 will homogeneously coat the surface. Third, BAX602 was developed as a surgical sealant for vascular anastomosis or lung surface leakage, and its adhesion prevention effect was discovered as an additional effect. Therefore, the adhesion prevention effects of BAX602 are not fully understood in clinical gynecological and abdominal surgical practice, while most other available adhesion prevention products have been used in gynecological and abdominal surgeries.

One may claim that the adhesion prevention effects of BAX602 will be more clearly proven by performing the second surgery at a longer time period than 2 weeks, such as several months or years after the first surgery. In fact, a previous randomized controlled study showed that BAX602 achieved significant adhesion prevention effects at 2–8 months after the first pediatric cardiac surgery [10]. In contrast, the present study will have a 2–12-week interval between the first and second cardiac surgeries, as the included patients are undergoing extracorporeal VAD implantation by the median sternotomy approach, and will need VAD explant surgery within 2–3 months [14]. However, the general adhesion process comprises fibrin network formation, inflammatory cell invasion, and then fibrosis that connects the tissues as adhesions. Of these processes, the degree of adhesion is largely determined by the second process (the invasion of inflammatory cells), which launches peak proangiogenic activities at 3–10 days postoperatively [8]. The BAX602 coating over the tissue surface inhibits the formation of fibrin networks that enable inflammatory cells to invade and settle, consequently reducing inflammatory cell invasion and the degree of adhesion formation. It is therefore reasonable to suggest that the adhesion prevention effects of BAX602 will be revealed as early as 2 weeks after the first surgery.

The scoring of the degree of adhesion based on macroscopic findings requires re-entry surgery, which is a major challenge in clinical studies evaluating post-surgical adhesion. As re-entry cardiac surgery is high-risk surgery, re-entry surgery only for the purpose of macroscopic assessment of the degree of adhesion would not be ethically permitted. However, the present study will include patients undergoing extracorporeal VAD implantation as bridge-to-decision therapy, which necessitates re-entry VAD explant surgery within a relatively short interval to either wean the patient from the VAD as a result of sufficient cardiac functional recovery or exchange the extracorporeal VAD with a durable VAD as a result of insufficient recovery [14]. Therefore, the present study enables the macroscopic soring of the degree of pericardial adhesion during VAD explant surgery.

There are other potentially useful endpoints in addition to the macroscopic findings. One good option may involve the use of imaging modalities such as cardiac magnetic resonance imaging or speckle tracking echocardiography, which do not require re-entry surgery; however, there is no established standard with which to quantitatively assess the degree of pericardial adhesion using these modalities. In addition, important information relating to adhesion formation could be gained from histological studies at 2–12 weeks after the first surgery, when the inflammatory cells would still be activated; however, tissue sampling from the cardiac surface for histological studies may induce complications such as bleeding, and thus would not be ethically permitted.

It may be advantageous to investigate the adhesion prevention effects of BAX602 in patients undergoing durable VAD implantation for bridge-to-transplantation, as these patients have a longer interval between surgeries than the present patient group. Animal studies that have reported non-clinical proof-of-concept for the adhesion prevention effects of BAX602 around the pericardium have used a longer interval between surgeries (12 and 25 weeks) than the interval in the present study [17, 18]; however, this did not include the implantation of an extracorporeal VAD [15]. As device implantation is likely to exacerbate the pericardial adhesion formation, the minimum intersurgery interval of 2 weeks in the present study will be sufficient to reveal the adhesion prevention effects of BAX602 in a statistically appropriate number of enrolled patients.

## References

[1] O'Brien SM, Shahian DM, Filardo G (2009). The Society of Thoracic Surgeons 2008 cardiac surgery risk models: part 2--isolated valve surgery. Ann Thorac Surg.

[2] Shahian DM, O'Brien SM, Filardo G (2009). The Society of Thoracic Surgeons 2008 cardiac surgery risk models: part 1--coronary artery bypass grafting surgery. Ann Thorac Surg.

[3] Shahian DM, O'Brien SM, Filardo G (2009). The Society of Thoracic Surgeons 2008 cardiac surgery risk models: part 3--valve plus coronary artery bypass grafting surgery. Ann Thorac Surg.

[4] Park CB, Suri RM, Burkhart HM (2010). Identifying patients at particular risk of injury during repeat sternotomy: analysis of 2555 cardiac reoperations. J Thorac Cardiovasc Surg.

[5] Mori M, Bin Mahmood SU, Schranz AJ (2020). Risk of reoperative valve surgery for endocarditis associated with drug use. J Thorac Cardiovasc Surg.

[6] Yoshitake S, Kinoshita O, Nawata K (2018). Single-center experience of the bridge-to-bridge strategy using the Nipro paracorporeal ventricular assist device. J Artif Organs.

[7] Leak LV, Ferrans VJ, Cohen SR, Eidbo EE, Jones M (1987). Animal model of acute pericarditis and its progression to pericardial fibrosis and adhesions: ultrastructural studies. Am J Anat.

[8] Moris D, Chakedis J, Rahnemai-Azar AA (2017). Postoperative abdominal adhesions: clinical significance and advances in prevention and management. J Gastrointest Surg.

[9] Gonzalez-Quintero VH, Cruz-Pachano FE (2009). Preventing adhesions in obstetric and gynecologic surgical procedures. Rev Obstet Gynecol.

[10] Hasaniya N, Razzouk A, Newcombe J (2018). An absorbable hydrogel spray reduces postoperative mediastinal adhesions after congenital heart surgery. Ann Thorac Surg.

[11] Pace Napoleone C, Valori A, Crupi G (2009). An observational study of CoSeal for the prevention of adhesions in pediatric cardiac surgery. Interact Cardiovasc Thorac Surg.

[12] Cannata A, Petrella D, Gambacorta M, Russo CF, Bruschi G, Martinelli L (2013). Histological findings following use of CoSeal in a patient with a left ventricular assist device. Surg Innov.

[13] Cannata A, Taglieri C, Russo CF, Bruschi G, Martinelli L (2009). Use of CoSeal in a patient with a left ventricular assist device. Ann Thorac Surg.

[14] Matsumoto M, Asaumi Y, Nakamura Y (2018). Clinical determinants of successful weaning from extracorporeal membrane oxygenation in patients with fulminant myocarditis. ESC Heart Fail.

[15] Davey AK, Maher PJ (2007). Surgical adhesions: a timely update, a great challenge for the future. J Minim Invasive Gynecol.

[16] Cannata A, Petrella D, Russo CF (2013). Postsurgical intrapericardial adhesions: mechanisms of formation and prevention. Ann Thorac Surg.

[17] Marc Hendrikx M, Mees U, Hill AC, Egbert B, Coker GT, Estridge TD (2001). Evaluation of a novel synthetic sealant for inhibition of cardiac adhesions and clinical experience in cardiac surgery procedures. Heart Surg Forum.

[18] Alizzi AM, Summers P, Boon VH, et al. Reduction of post-surgical pericardial adhesions using a pig model. Heart Lung Circ. 2012;21:22–9. 10.1016/j.hlc.2011.10.002 22078313

